# Biology-Culture Co-evolution in Finite Populations

**DOI:** 10.1038/s41598-017-18928-0

**Published:** 2018-01-19

**Authors:** Bart de Boer, Bill Thompson

**Affiliations:** 1 Artificial Intelligence Lab, Vrije Universiteit Brussel, Pleinlaan 2, B-1050 Brussels, Belgium; 2 Language and Cognition Department, Max Planck Institute for Psycholinguistics, Wundtlaan 1, 6525 XD Nijmegen, The Netherlands

## Abstract

Language is the result of two concurrent evolutionary processes: biological and cultural inheritance. An influential evolutionary hypothesis known as the moving target problem implies inherent limitations on the interactions between our two inheritance streams that result from a difference in pace: the speed of cultural evolution is thought to rule out cognitive adaptation to culturally evolving aspects of language. We examine this hypothesis formally by casting it as as a problem of adaptation in time-varying environments. We present a mathematical model of biology-culture co-evolution in finite populations: a generalisation of the Moran process, treating co-evolution as coupled non-independent Markov processes, providing a general formulation of the moving target hypothesis in precise probabilistic terms. Rapidly varying culture decreases the probability of biological adaptation. However, we show that this effect declines with population size and with stronger links between biology and culture: in realistically sized finite populations, stochastic effects can carry cognitive specialisations to fixation in the face of variable culture, especially if the effects of those specialisations are amplified through cultural evolution. These results support the view that language arises from interactions between our two major inheritance streams, rather than from one primary evolutionary process that dominates another.

## Introduction

The human proclivity to learn, process, and deploy natural language is unique among the animal kingdom, yet universal in our species and central to human life and survival, pointing to a unique biological endowment for these skills. How could such an endowment have evolved? Much disagreement surrounds this question. Orthodoxy argues that the evolution of a unique language-ready brain is, like any other biological trait, explainable unproblematically in hyper-adaptationist terms: any feature of language that improves communication also improves the inclusive fitness of any individual who is better prepared for this feature; as a result, biological evolution is thought to have shaped in us a rich suite of idiosyncratic, domain-specific cognitive biases that underpin our linguistic abilities^1^.

However, this view is being challenged by growing support for the alternative view that many of the defining characteristics of language could have arisen through *cultural* evolution^2,3^: as language is transmitted to new learners^4^, and deployed for communication^5,6^, pressures acting on these cultural processes drive the emergence of linguistic structure^7^. This perspective is sometimes taken to imply an opposing understanding of the role of biological evolution in shaping human language. In its most extreme form, a focus on the primacy of cultural evolution has led some to question not only whether biological evolution of a specialised language faculty is the *better* explanation of human language, but also whether a biological account is even coherent: “… a biological endowment could not coevolve with properties of language that began as learned cultural conventions, because cultural conventions change much more rapidly than genes… this rules out the possibility that arbitrary properties of language, including abstract syntactic principles governing phrase structure, case marking, and agreement, have been built into a “language module” by natural selection. The genetic basis of human language acquisition and processing did not coevolve with language…”^8^.

These two explanations for language, and the evolutionary processes they respectively imply, are intrinsically linked at a mechanistic level: cultural evolution is powered by repeated learning, transmission, and use, which are all underpinned by biologically evolved cognitive mechanisms; biological evolution is shaped by inclusive fitness, which – in a radically social species like ours – is a function of culturally transmitted traits and their distribution among populations^9^. This dependence implies a *co-evolutionary* process underpinning the emergence of language in our species^10,11^: this class of processes is central to human evolution^12^, yet the implications for language and cognition^13^ are only beginning to be understood formally^8,14,15^. At present, the available toolkit for studying biology-culture co-evolution is limited in fundamental ways. Here, we address one such limitation: the absence of a mathematical framework for studying biology-culture co-evolution in realistically sized finite populations, and the stochastic effects that are so crucial to their evolution (although see chapter 2 of^16^ for early modeling of gene-culture co-evolution in small finite populations: our model can be seen as a natural extension of these earlier frameworks from biology).

Evolution in variable environments has long been a topic of interest in population biology^17^, and has recently received renewed attention^18–24^. This literature provides a rich mathematical backdrop for exploring these questions, but tends to focus on the behaviour of evolutionary processes in the abstract, rather than addressing specific questions about the evolution of human language and cognition. A small but influential literature on specifically language-related co-evolutionary questions has driven several debates in the language sciences. For example, Nowak *et al*.^25^ mathematically confirmed Pinker and Bloom’s^1^ influential suggestion that selection for linguistic coordination favors the emergence of learners who are innately specialized to acquire an arbitrary subset of possible languages. Other models have led to similar conclusions: for instance, Briscoe^26^ reasons that, in finite populations, only a subset of possible languages can ever be employed – as such, any cognitive specialization toward acquiring that subset faster or more reliably is beneficial to the learner and will be selected for, suggesting that the finite nature of real populations may inherently influence the evolution of cognition.

More recently, these dynamics have been questioned by evolutionary models that focus on rapid cultural change, or the moving target problem^8,24,27,28^. These models illustrate the claim that there are essential limitations on cognitive adaptation to culturally transmitted language, and that these limitations result from the *rate* of cultural evolution. Limitations of this kind, which govern the interactions between our two major inheritance streams, would be of fundamental importance to our understanding of human evolution, yet relatively little is known about the conditions under which the moving target problem holds. For example, one well understood aspect of cultural evolution is its capacity to amplify the effects of cognitive biases^9,29^. Recent evolutionary analyses^15,30,31^ suggest that, as a result of this aspect of culture, minor cognitive tweaks that impose weak inductive biases can rapidly spread through a population: in this way, culture can perhaps even *speed up* cognitive adaptation, thanks to the amplifying feedback processes that link culture and biology. These analyses are not tailored to the problem of rapidly varying culture, but nevertheless suggest a tempting hypothesis about this case: amplification dynamics may be key to where the moving target problem can apply.

It is currently unclear how these two lines of reasoning (rapid change & bias amplification) about culture interact, since no model includes both. More generally, all the models of language evolution reviewed above are limited by one of two methodological restrictions which we aim to relax: either they are mathematically precise, but consider only the limiting case of infinitely large populations and cannot capture the stochastic processes that shape finite populations;^15,24,25,31,32^ or they consider finite populations, but are implemented as agent-based simulations that cannot be comprehensively analysed and contain many ad hoc design decisions^8,15,26–28,33^. Since it can be assumed that ancestral hominin populations were small (effective ancestral population size has been estimated at 12800, with bottlenecks down to 600 individuals^34^; average ethno-linguistic group size of hunter-gatherers is about 1500^35^), stochastic effects may well have been key to the persistence of any improbable cognitive changes that underpin language. Infinitely-large-population models cannot naturally capture these effects.

These considerations lead to a clear goal that is currently unachievable: the ability to explicitly state the probability of co-evolutionary outcomes in finite populations, taking stochastic effects and feedback processes between biology and culture into account. Our framework makes this possible. We present a generalization of the Moran Process^36^ - a classic mathematical framework for studying stochastic evolution in finite populations - to include time-varying culture which is dependent upon the biological population. The Moran process models a population of fixed size that consists of two biological variants (to be concrete, let us assume linguistic specialists and generalists). It has a state for each number of specialists (from zero to the population size) and allows adding or subtracting one specialist (or keeping the same number) per time step. We extend the Moran process by splitting each biological state into a number of states equal to an arbitrary number of cultural states, and we allow not only adding or subtracting specialists through biological evolution, but also transitions between cultural states to model cultural evolution. Probabilities of transitions between biological states may depend on the cultural state and probabilities of transitions between cultural states may depend on the biological state. Conceptual and mathematical detail is found in the methods section.

The framework allows us to gain mathematically precise insights into the evolution of specialisation for cultural behaviours among small, finite populations of precisely the kind in which language must have emerged. We focus on two key ideas to emerge from the language evolution literature, both thought to substantially re-order co-evolutionary dynamics: relative *speed of change* arguments, which underpin the moving target problem;^8^ and *amplification dynamics*, whereby cultural evolution is thought to amplify the effects of cognitive biases^15,30^. Our analysis shows that the moving target effect declines with population size and with increased amplification. Even when culture strongly out-paces biology, there are realistic conditions that allow *co-evolution* to build behaviour.

## Results

We used our approach to investigate a model designed to correspond closely to existing simulations of the moving target problem^8^. The model captures a biological population whose fitness depends upon the ability to acquire a cultural trait that varies rapidly between two arbitrarily distinct alternatives (our approach allows for an arbitrary number of cultural states, but here we analyse the simplest possible case). The population is comprised of generalists, who can acquire either cultural trait equally well, and specialists, who are better at learning one of the two states but worse at learning the other. The model can be used to study the effects of population size, fitness differences, speed of change, amplification, and initial conditions. We focus on three core issues: comparison of the cultural and non-cultural versions of the model; the speed of cultural change; and amplification dynamics. In each case, our analyses ask how likely is a single mutant specialist to take over a population of generalists (fixation probabilities), and how rapidly that could happen (conditional fixation times). Full details of the model are given in Methods.

### The Effects of Culture

**Figure 1:**
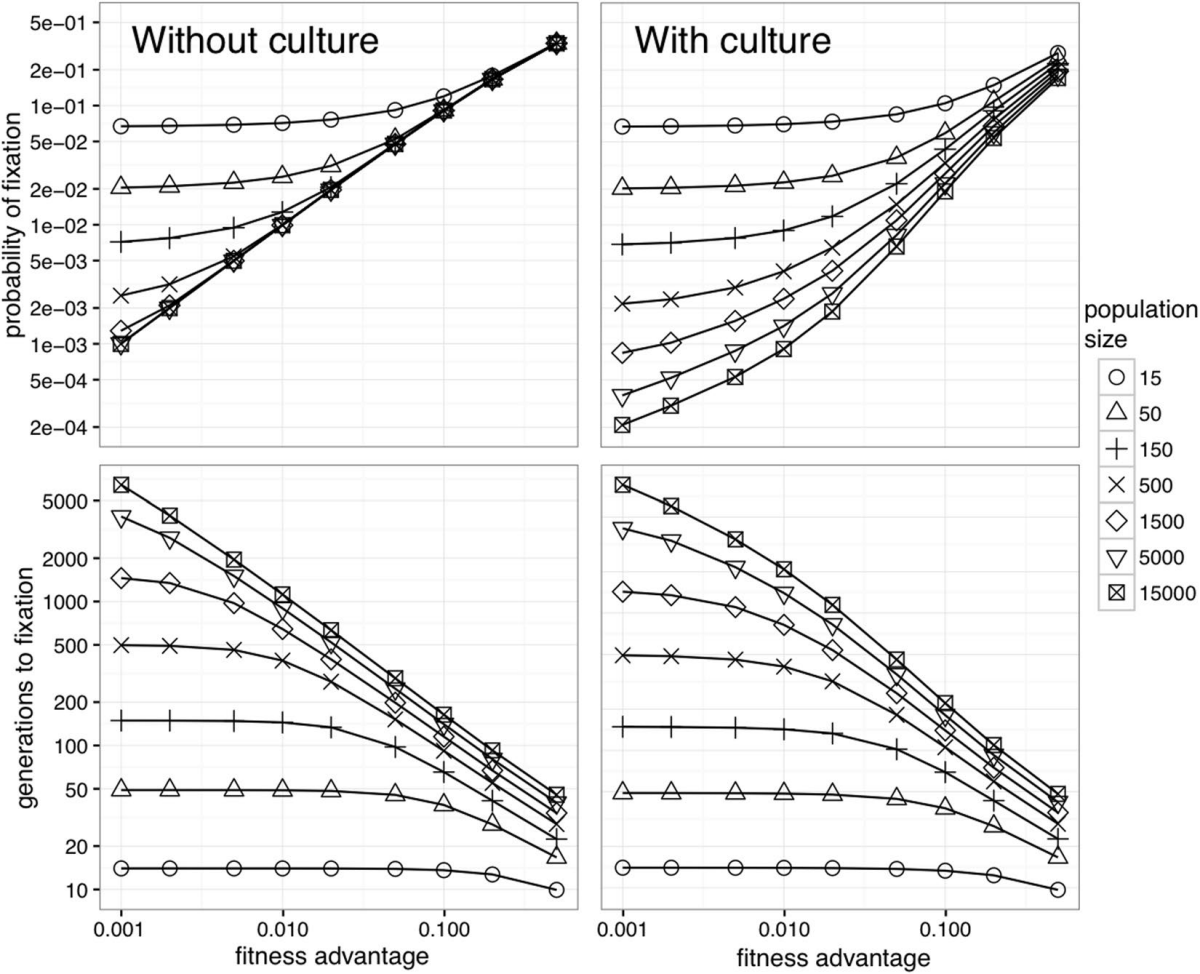
Fixation probabilities (top) and times (bottom) for populations without culture (left) and with culture (right).

Figure 1 shows fixation probabilities (top) and times (bottom), as a function of population size and the difference in fitness between generalists and specialists, in the standard Moran process (without culture, left) and our co-evolutionary generalisation (with culture, right) under matched parameter settings (sensible default values: *η* = 0.1 means that culture changes roughly once every ten generations; and *α* = *β* = 1 are suitable neutral values for equation 9 which determines the degree of amplification). The coupling between the biological population and cultural change is determined by *α* and *β*. We will introduce these more precisely in the section on amplification below, where we will also study the effect of varying them. A mathematical definition is provided in the methods section, equation 9. The cultural system starts in the preferred state (though see Supplementary Information S.2 for alternative assumptions). It is clear that, in larger populations, fixation probabilities are lower when the model includes cultural evolution, consistent with the moving target argument and existing results^8^. However, in smaller populations, where stochastic effects play a greater role, these differences between the cultural and non-cultural model are notably reduced: in realistically sized finite populations, cultural change is less of an obstacle to cognitive specialisation.

### Speed of Cultural Change

**Figure 2:**
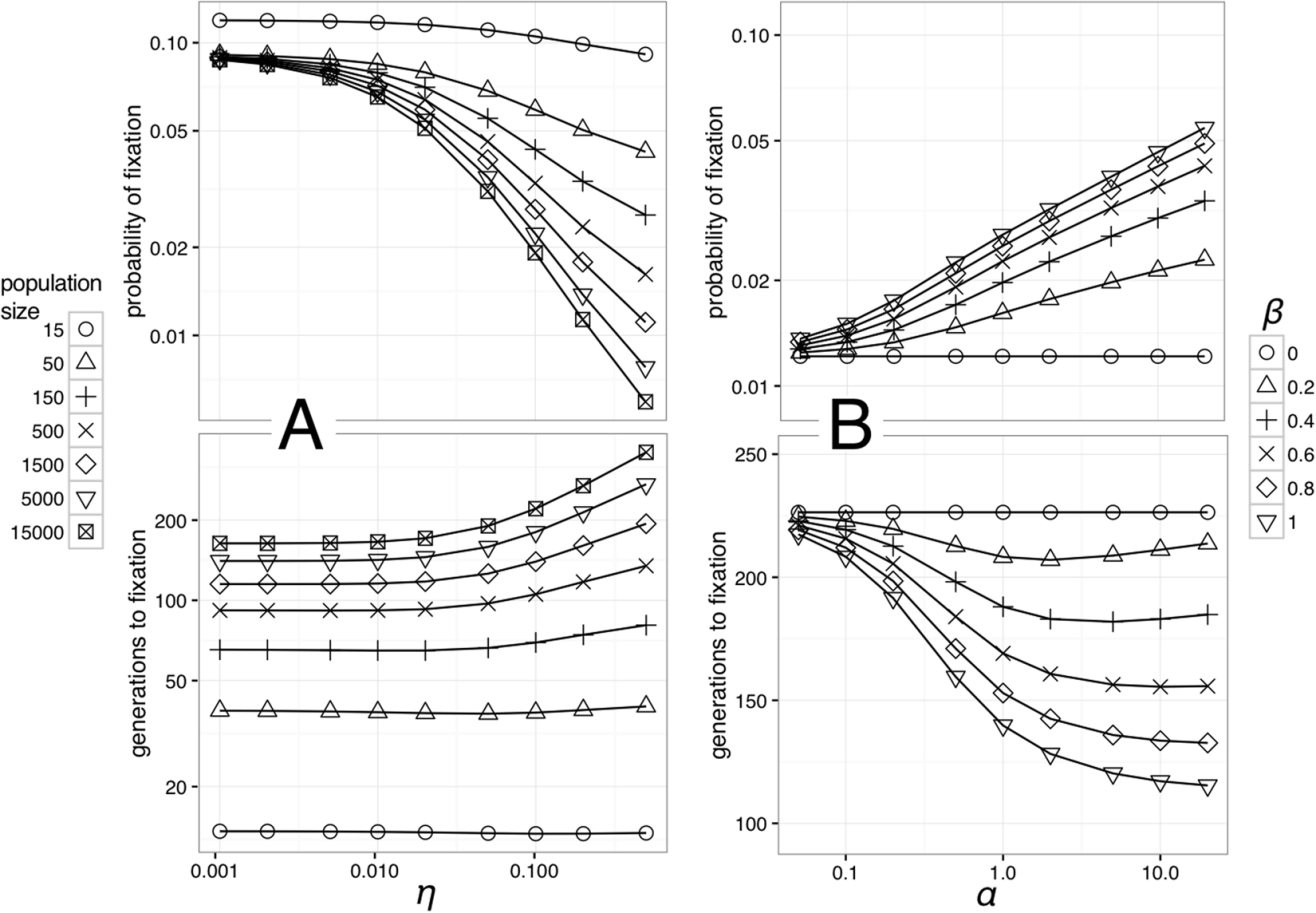
Fixation proabilities (top) and times (bottom) as a function of the rate of cultural change (**A**, left) and the degree of amplification (**B**, right). In (**A**), no amplification is assumed: *α* = *β* = 1. Faster cultural change reduces the probability of adaptation, but much less so in smaller populations. In (**B**), the rate of cultural change is held steady at *η* = 0.1 and the population size is 1500. Greater amplification (larger *α*,*β*) increases fixation probability. In both, a small fitness difference is assumed: *φ* = 0.1.

Figure 2A shows fixation probabilities and times as a function of the rate of cultural change, *η*, (which specifies the probability of culture changing per generation, i.e. for *η* = 0.1, culture is expected to change every ten generations). Across the board, faster cultural change (higher *η*) results in lower fixation probabilities and greater fixation times, as is implied by the moving target problem. The supplementary material section S.4 contains graphs that directly show the amount by which culture affects the rates and probabilities of change: these graphs show the ratios between the probabilities and fixation times with and without culture. However, two qualifications to this pattern are clear. First: in realistically small populations with a moderate rate of cultural change, fixation probabilities are not much lower than for no cultural change. For instance for population size *N* = 500 and rate of cultural change $\eta =0.1$, the fixation probability is only about one third of the fixation probability without culture. Second, the moving target problem is clearly dependent on population size: in smaller populations, fitness differences are relatively less important than in larger populations. The effect of the intermediate fitness differences appears to be largest: for small fitness differences, drift dominates, for large fitness differences, biological evolution converges so fast the cultural changes have little effect. For intermediate fitness differences, biological evolution is sufficiently slower than cultural evolution for them to interfere with each other. The effect of cultural change is that more rapid cultural change leads to slower and less probable fixation. However, only for very large population sizes does this become an issue, and even then it turns out that for a population of 15 000 individuals, and a culture that changes every 2 generations, the probability of fixation is reduced approximately ten-fold and the time to fixation 2-fold. Amplification is important however: the likelihood of cultural states should be influenced strongly by the presence of only a few specialists. Still, even this effect is small. If no amplification is present, fixation probabilities are reduced 8-fold and fixation times increased 2-fold compared to no culture at all. In sum, rapid cultural change is an obstacle to specialisation, but not a roadblock, and its consequences are contingent on other factors, not least population size.

### Amplification

Figure 2B shows fixation probabilities and times as a function of our assumptions about the degree of amplification: are specialists more likely to take over a population if their presence biases the direction of cultural change?

When the population consists only of *generalists*, the culture spends half of its time in each cultural state (because both states are equivalent). However, when the number of specialists grows, it is logical that the culture will spend more time in the state that the specialists prefer, because for instance this state is easier for them to acquire. This growth in time spent could be proportional to the proportion of specialists, but it is also possible that initially it grows faster than the proportion of specialists, or slower. This is referred to as the level of amplification, and it is modeled by parameter *α*. A value of 1 corresponds to proportional growth, *α* > 1 corresponds to amplification and 0 ≤ *α* ≤ 1 corresponds to the opposite.

When the population consists only of *specialists*, it is not necessary that the culture always corresponds to their preferred state. It is of course possible that the culture is always in the preferred state, but it is equally possible that it still fluctuates. This is determined by parameter *β*. If *β* = 1, the culture always is in the preferred state if the population consists only of specialists, but if *β* = 0, the culture still spends 50% of its time in each state, independent of population composition. The particular way this is done in the model is discussed in the methods section, and formalized in equation (9).

Increasing *β* results in increasing fixation probability and decreasing fixation time. This is to be expected, because a higher value of *β* means the population spends a larger proportion of time in the preferred cultural state for any given population composition. A similar amplification effect results from increasing *α*, which controls the relationship between the number of specialists in the population and their influence on cultural change. Increasing *α* results in increased fixation probability and decreased fixation time. Amplification allows cognitive specialisation to have a disproportionate influence on the state of the culture: in this setting, it allows specialists to counteract the moving target problem by ensuring cultural change proceeds towards favoured states.

## Discussion

The presence of a culture that switches between two states reduces the probability that specialists will take over a population of generalists, but less dramatically than predicted by previous models^8^. The moving target problem is notably less problematic in small populations, where stochastic effects can dilute the importance of fitness differences. Moreover, the relation between the proportion of specialists in the population and the proportion of time spent in the preferred cultural state is crucial. If this relation is amplifying, fixation probabilities and fixation times are closer to those of evolution under a culture-independent fitness advantage. Taken together with existing results^15,30^, our findings suggest that this kind of relationship - which we have called amplification dynamics - can have an important impact on cognitive evolution. If, where, and how these dynamics play out in cultural evolution is therefore a key empirical question.

The model highlights an important subtlety in co-evolutionary dynamics. If cognitive specialisation leads to benefits when acquiring the favoured cultural variant, but entails equivalent costs for acquiring the alternative variant (as it does in the model), why do specialists gain an overall fitness advantage? The reason specialists nevertheless take over with fixation probabilities larger than those under drift is this: once a few specialists are established in the population, the culture will spend more time in the preferred state, thus effectively giving the specialists a fitness advantage. This advantage increases with an increasing number of specialists. This result is a direct consequence of the feedback processes between biological and cultural evolution – the *level-dependence* we introduced. Note that infinite population models would not capture this dynamic, which depends on random drift in the early stages. Our findings also contrast with Chater *et al*.’s *finite* population simulations. In that model, specialists have a much stronger disadvantage in the dispreferred cultural state than the advantage they enjoy in the preferred state (see Supplementary Information), an assumption we relaxed.

While our analyses are tailored towards versions of the model that are directly comparable with existing models^8^, there are a number of abstractions in this framework whose implications are worth noting. For example, the Moran model we extend does not accommodate many important aspects of evolution, such as sexual recombination, variable population sizes, or mutation. More complex models that incorporate features such as these will be valuable extensions of this work. Likewise, there are aspects of real linguistic populations that are not accounted for in our model or comparable existing models. One such factor is the difference in scale between genetic and linguistic diversity ancestrally: particularly in small populations, a reasonable objection to our framework is that linguistic diversity in a population far outweighs genetic diversity. This discrepancy surely has consequences for co-evolutionary dynamics, and this would be a valuable subject for future analyses.

Another important aspect of the design decisions on which our model is constructed concerns the nature of the cognitive mechanisms under study, and their role in driving cultural evolution. By design, our analysis does not directly model processes of observation, inference, induction, reasoning, and production that underpin cultural transmission of complex traits like language. We pursue a different level of analysis, directly modeling transition probabilities between cultural states, and conditioning these transitions on the balance of specialists and generalists in the population (as well as on the previous cultural state and an externally driven rate of fluctuation). In this respect, the model abstracts over the nature of the specialists. Other models do examine co-evolution under specific assumptions about the cognitive processes underpinning cultural transmission via inductive inference^15^, and we are enthusiastic about extending this approach to the case of rapidly-varying culture in the future. However, our model does concern a specific class of cognitive mechanisms: those that favour particular cultural variants, or categories of cultural variant, rather than, for example, improvements to learning that are variant-neutral (such as an inclination toward high-fidelity imitation, for instance). In the traditional cultural evolution literature, this kind of cognitive adaptation is known as a direct bias^9^. In contemporary terms, this kind of adaptation can be thought of as an inductive bias that implies a prior distribution over variants at the lowest level of abstraction: low-level biases for specific constituent ordering patterns, morpho-syntactic strategies or representational formats, and phonetic or articulatory variants are all examples of the kinds of cognitive adaptation this model concerns. Our analysis of the general case encourages deeper investigation of specific cases: we have shown that speed-of-change arguments do not rule out the evolution of these kinds of biases a priori.

In summary, the model presented here represents a step towards a better mathematical understanding of an evolutionary processes that is thought to be central to human uniqueness: biology-culture co-evolution in finite populations. Under realistic assumptions - populations of around 1000 individuals and culture that changes every few generations - biological adaptations to culture can become fixed, especially when the relation between the number of specialists in the population and the amount of time spent in the preferred cultural state is amplifying. There are conditions under which the pace of cultural evolution does not limit behaviour-building interactions between biological and cultural inheritance streams.

## Methods: Model Description

The model is based on the Moran process^36,37^. The Moran process describes change in the composition of a finite, fixed-sized population in which two types (alleles) compete. The state of a population with *N* individuals is fully described by the number *i* = 1, …, *N* of one of the two types of individuals (the number of the other type being *N* − *i*). Time is discrete: at each time step one individual is selected to procreate (by creating a copy of itself) and one individual is selected to be removed from the population. The same individual can be selected in both cases. At each time step the number of individuals of a given type can increase or decrease by maximally one, or remain unchanged. The transition probabilities between states are:

1 $$P(i+1|i)=\frac{N-i}{N}\cdot \frac{(1+{f}_{i})i}{N+{f}_{i}\cdot i},$$

2 $$P(i-1|i)=\frac{i}{N}\cdot \frac{N-i}{N+{f}_{i}\cdot i},$$

and *P*(*i*|*i*) = 1 − *P*(*i* + 1|*i*) − *P*(*i* + 1|*i*), where *f*_*i*_ is the fitness advantage (or disadvantage if negative) of the indexed type. The advantage of the Moran process over less restricted (e.g. any number of individuals can be replaced) models of population change in finite populations is the simplicity of its mathematical structure: the process can be described as a linear Markov chain and modeled as a tridiagonal matrix of transition probabilities between states. This form of matrix can be efficiently solved numerically, and solved analytically in special cases^37,38^. We focus on two properties of the model: fixation probability and mean conditional fixation time (for a given *i*). The fixation probability is the probability that the population ends up in the state $i=N$, where all individuals are of the same type. Conditional fixation time is the mean time taken for the population to reach the state $i=N$, given that the population does reach that state. Of particular interest are the fixation probability and the fixation time for starting in the state $i=1$: these are, respectively, the probability that a single mutant will take over the population, and the expected time that this will take.

### The Moran Process with Culture

In the standard Moran process, fitness is a fixed function of the biological population composition. Here we wish to relax this assumption, and instead capture the idea that fitness benefits are accrued by acquiring a cultural behaviour that, like language, can take a range of forms and varies over time (fitness benefits have been proposed to result from conventional language in a number of ways, such as communicative alignment^39^, or producing and or comprehending shared signals more generally^40^, or as a marker of group membership^41^ - the model is designed to generalise over these specific mechanisms). This brings our analysis closer to the biological literature: our model is formally closest to that of Ashcroft *et al*.^21^, who also model adaptation to variable environments in finite populations. Our analysis departs from theirs, and from the varying-environment literature more generally, by our assumption that the composition of the biological population influences how the environment – in our case culture - varies over time. In technical terms, this is known as *level-dependence*: intuitively, it introduces a feedback loop between the two inheritance streams. This feature significantly complicates the model and its analysis, but is an essential part of biology-culture co-evolution, and will turn out to be paramount to the evolutionary dynamics.

We assume that, at any given point in time, the culture of the population is in one of *k* possible states, corresponding to *k* types of language. To capture this assumption, each state of the population must be split into as many states as there are different types of culture. Where there are *i* individuals of the indexed type, and the culture is in state *c*, we will say the system is in state *s*_*i*,*c*_. Transition probabilities between states *s*_*i*,*c*_ and *s*_*j*,*c*′_ (for *j* = 0, …, *N* and *c*′ = 1, …, *k*) are given by *P*(*j*,*c*′|*i*,*c*), and must be zero if |*j* − *i*| 1. This is the condition of the Moran process that only one individual gets replaced at a time. There is no analogous restriction on transitions between cultural states.

**Figure 3:**
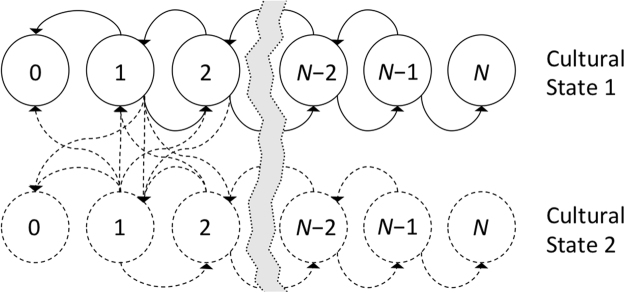
The Moran process (with culture) as a Markov chain. The upper chain (solid lines) illustrates an ordinary Moran process. The dashed states indicate the states that must be added in a model with two cultural states. For clarity, only the extra transitions from and to states with one indexed individual are given, but for the complete Markov chain, extra transitions must be added for all states.

**Figure 4:**
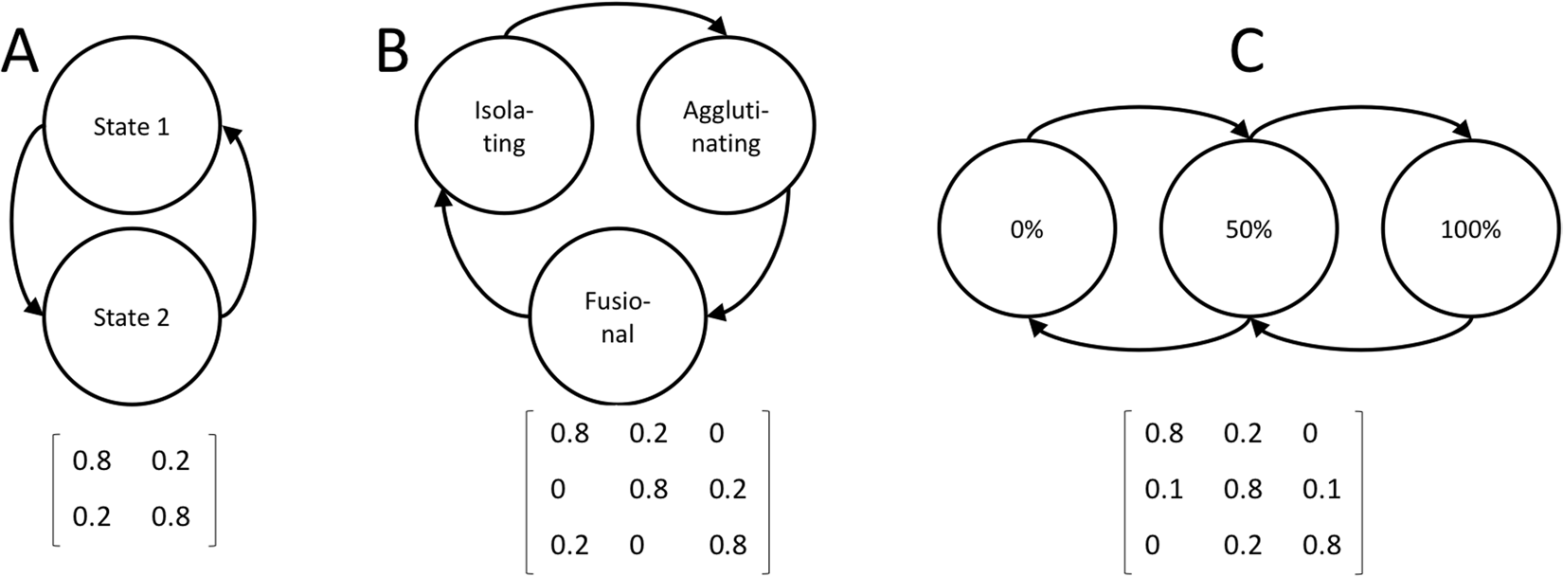
Examples of cultural transition graphs and transition matrices, illustrating ways in which biological states could be split up. Please note that for clarity’s sake, transitions to other biological states and from states to themselves are not drawn. (**A**) The simplest possible model with two cultural states, similar to the example model analyzed in the paper. (**B**) Situation with three cultural states that can change in one direction, but not the other, inspired by the proposed historical change of types of morphology. (**C**) Transition graph for modeling intermediate cultural states, where there are two cultural states and the culture can be pure (the 0% and 100% states) or an intermediate mixed state (the 50% state). The transition matrices would be used to build up the block-tridiagonal matrix of the final model. It should be noted that the transition matrices in the final matrix would also depend on the biological composition of the population.

The addition of culture with two states leads to the Markov process illustrated in Fig. 3. Alternative ways to transition between cultural states are illustrated in Fig. 4, which also provides a linguistic example. A population of *N* individuals and *k* cultural states can be formulated as a (*N* + 1)*k* × (*N* + 1)*k* block tridiagonal matrix (analogous to a tridiagonal matrix, but with *k* × *k* blocks rather than numbers on the three diagonals). The transition probabilities can be simplified by assuming that cultural change and biological change are seperate events, giving:

3 $$P(j,c\text{'}|i,c)=P(j|i,c)P(c\text{'}|i,c)$$

where *P*(*j*|*i*,*c*) is the probability of ending up with *j* individuals of the indexed type when in a population that contains *i* individuals of that type and is in cultural state *c*. The probability *P*(*c*′|*i*,*c*) is the probability of going from cultural state *c* to cultural state *c*′ when there are *i* individuals of the indexed type in the population.

### Calculating fixation probability and fixation time

The procedure for calculating the fixation probabilities of an ordinary Moran process is a well-established textbook method^37^. Following the notation of Antal and Scheuring^38^ (their eq. 5), the probability of ending up with a population that contains only individuals of the indexed type, when starting with *i* individuals of that type, can be denoted by *ε*_*i*_ and computed with (4):

4 $${\varepsilon }_{i}={\mu }_{i}{\varepsilon }_{i-1}+(1-{\mu }_{i}-{\lambda }_{i}){\varepsilon }_{i}+{\lambda }_{i}{\varepsilon }_{i+1}$$

where *μ*_*i*_ = *P*(*i* − 1|*i*) and *λ*_*i*_ = *P*(*i* + 1|*i*) are transition probabilities given by equations (1) and (2) respectively, and by definition *ε*_0_ = 0 and *ε*_*N*_ = 1. These equations can be solved analytically using what is in essence an application of the Thomas algorithm^42^. An expression analogous to (4) can be given for fixation probabilities in the Moran process with culture:

5 $${\vec{\varepsilon }}_{i}=\,{\rm{M}}{}_{i}{\vec{\varepsilon }}_{i-1}+\,{\rm{K}}{}_{i}{\vec{\varepsilon }}_{i}+{{\rm{\Lambda }}}_{i}{\vec{\varepsilon }}_{i+1}$$

where  $\mathop{{\varepsilon }_{i}}\limits^{\longrightarrow}$= (*ε*_*i*,1_, …, *ε*_*i*,*k*_)^*T*^ is a vector of probabilities: each element *ε*_*i*,*c*_ gives the probability that the indexed type will take over the population if initially there are *i* individuals of that type and the culture is in state *c*. Again, $\mathop{{\varepsilon }_{0}}\limits^{\longrightarrow}$ = (0, …, 0)^T^ and $\mathop{{\varepsilon }_{{\rm{N}}}}\limits^{\longrightarrow}$ = (1, …, 1)^T^ by definition. M_i_, K_i_, and Λ_*i*_ are the matrices of transition probabilities between all cultural states while respectively losing one individual of the indexed type (M_i_), keeping the same number (K_i_), and increasing by one (Λ_*i*_). In the limit of a single unchanging cultural state, these equal the terms in equation (4): the limit *κ*_*i*_ → 1 − *μ*_*i*_ − *λ*_*i*_ obtains because the probabilities of leaving a state must sum to one.

Antal and Scheuring^38^ also provide expressions (their equations 13 and 14) for calculating the fixation *time*. Their derivation can be generalised to our system with multiple cultural states. If $\mathop{{\tau }_{0}}\limits^{\longrightarrow}$ = (*τ*_*i*,1_, …, *τ*_*i*,*k*_) is a vector of mean fixation times, from each cultural state, assuming an initial population with *i* indexed individuals, then we can write:

6 $$-{\vec{\varepsilon }}_{i}={\rm{M}}{}_{i}{\vec{\tau }}_{i-1}+(\,{\rm{K}}{}_{i}-{\rm{I}}\,){\vec{\tau }}_{i}+{{\rm{\Lambda }}}_{i}{\vec{\tau }}_{i+1},$$

where I is the identity matrix. This allows us to compute the *conditional* fixation time: specifically, the conditional fixation time, which we denote *t*_*i*,*c*_, describes the the average time it will take for a population to go to fixation, conditional on the fact that the indexed type does indeed take over the population. Noting that $\mathop{{\tau }_{0}}\limits^{\longrightarrow}$  = (0, …, 0)^T^ and  = $\mathop{{\tau }_{0}}\limits^{\longrightarrow}$ (0, …, 0)^T^, conditional fixation times in the cultural model are given by *t*_*i*,*c*_ = *τ*_*i*,*c*_/*ε*_*i*,*c*_.

The system of equations defined by (6) is sparse, and can therefore be solved numerically in an efficient way.

### Application to language evolution

For direct comparison with existing results, we tailor our analysis to the case explored by Chater *et al*.^8^. While their simulations assume learners are adapting to multiple linguistic behaviours, those behaviours (and the genes underpinning them) are independent, so the question can be collapsed to a single behaviour without loss of generality. On these terms, their model has two cultural states (*k* = 2), which stand for two arbitrarily distinct types of language. Though this case is distantly removed form the richness of natural language, we believe it represents the right level of abstraction when beginning to explore these questions: more complex models remain distantly removed from reality, and quickly become extremely difficult to interpret; asbtract models have proven to be a productive method for distilling general arguments into test cases that nevertheless generalise and can be widely discussed^8,15,29,31,43^. Our model can be extended beyond the two-language case, and could even capture structured models of cultural types and their evolutionary dynamics, by imposing this structure on M_i_, K_i_, and Λ_*i*_. We leave these extentions for the future.

The population consists of specialists (the indexed type) and generalists (the other type). Generalists are equally good at learning both types of language. Specialists are better at learning one type (i.e. acquire it faster, more reliably, or from fewer examples), but worse at learning the other. The state at which the specialists are better is called the preferred state, and denoted with +; the other state is denoted with −. Following Chater, Reali, and Christiansen^8^, fitness reflects the ability to learn the *correct* language: in other words, whichever language the population at large is using. Also following Chater *et al*.^8^, the two language types are arbitrarily distinct, in the sense that neither has an intrinsic advantage over the other (but see Supplementary Information S.3 for an example of what happens when this is not the case). Therefore, the disadvantage of the specialists in one state of culture is equal to their advantage in the other state:

$${f}_{s}=(\begin{array}{cc}1+\varphi  & {\rm{in}}\,{\rm{the}}\,{\rm{preferred}}\,{\rm{state}}\\ 1-\varphi  & {\rm{in}}\,{\rm{the}}\,{\rm{other}}\,{\rm{state}},\end{array}$$

and *f*_*g*_ = 1, where *f*_*g*_ and *f*_*s*_ give the fitness of generalists and specialists respectively, and *φ* denotes a small fitness difference between the two. We can set *f*_*g*_ = 1 without loss of generality, as only the ratio between *f*_*g*_ and *f*_*s*_ is important. Substituting these into (1) and (2), we obtain:

7 $$P(i+1|i,\pm )=\frac{N-i}{N}\cdot \frac{(1\pm \varphi )i}{N\pm \varphi \cdot i}$$

8 $$P(i-1|i,\pm )=\frac{i}{N}\cdot \frac{N-i}{N\pm \varphi \cdot i},$$

and, similarly, *P*(*i*|*i*, ±) = 1 − *P*(*i* + 1|*i*, ±) − *P*(*i* + 1|*i*, ±). In these equations, we choose all + signs when transitioning from the preferred state and all – signs from the other state.

### Amplification Dynamics

**Figure 5:**
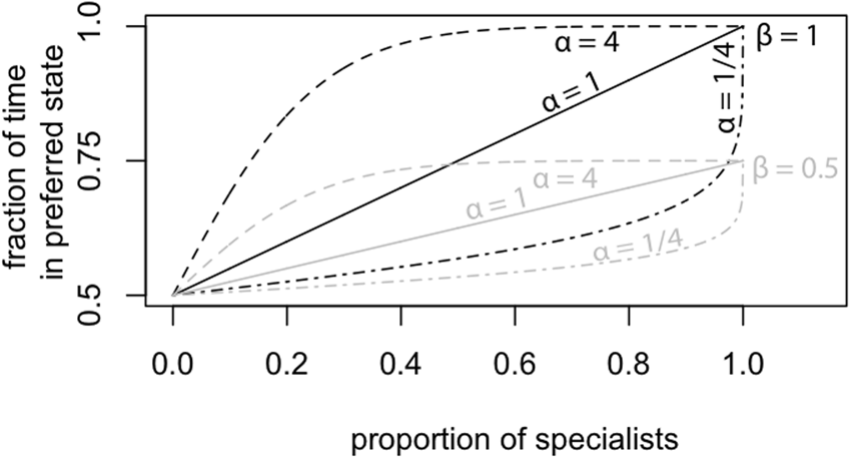
Illustrations of the function (see eq. 9) used to convert the proportion of specialists in a population to the fraction of time spent in the state preferred by the specialists.

So far we have said nothing of how the biological composition of the population relates to the cultural state of the population. Feedback between cultural and biological evolution is captured by allowing the probability of transitioning from one cultural state to another to depend on the composition of the population. When specialists are more prevalent, cultural evolution will favour the preferred language type, leading the population to spend more time in that state. As by definition both cultural states are equivalent, the population will spend half of its time in each cultural state when there are only generalists. There are infinitely many functions that fulfil these conditions. Related models have focused specifically on these relations, deriving them directly from particular psychological models of language learning and production^15,29,30^. Here we aim instead for generality, but for concreteness’s sake a family of representative functions is given by:

9 $$\pi (x)=\beta \frac{{(1+x)}^{\alpha }}{{(1+x)}^{\alpha }+{(1-x)}^{\alpha }}+\frac{1-\beta }{2},$$

where *π*(*x*) is the fraction of time the population spends in the preferred cultural state if there is a proportion 0 ≤ *x* ≤ 1 of specialists in the population. Here *α* is a positive real number that determines the steepness of the relation. If *α* is larger than one, the proportion of time spent in the preferred state rises rapidly when only a small number of specialists is present; if *α* is between zero and one, this proportion rises slowly when a few specialists are present. Similarly, *β* determines the fraction of time spent in the preferred state when the population consists of specialists exclusively. If *β* is zero, the fraction is 1/2; if *β* is one, the fraction is one. Figure 5 shows the influence of these parameters.

### Speed of Cultural Change

The fraction of time spent in each cultural state does not fully determine the transition probabilities. It is possible to obtain the same fraction with very low transition probabilities (slowly changing culture) or with higher transition probabilities (rapidly changing culture). The transition probabilities are:

10 $$P(+|-)=\frac{\eta }{N}\cdot \pi (\frac{i}{N})$$

11 $$P(-|+)=\frac{\eta }{N}[1-\pi (\frac{i}{N})]$$

where *η* is the parameter that determines the speed of cultural change. Larger *η* corresponds to faster cultural change. The complete equations of the model can be found in Supplementary Information S.1.

## Electronic supplementary material

Supplementary Information
