# Supplementary material for: Biology-Culture Co-evolution in Finite Populations
Source: Sci Rep. 2018 Jan 19;8:1209. doi: 10.1038/s41598-017-18928-0 (PMC5775438; doi:10.1038/s41598-017-18928-0)
Supplement: Supplementary file 1 — Supplementary Information [file 41598_2017_18928_MOESM1_ESM.pdf]

Biology-Culture Co-evolution in Finite Populations
Supplementary Information

Bart de Boer[1,2]
Bill Thompson[1,2]

[1] Artificial Intelligence Lab, Vrije Universiteit Brussel, Pleinlaan 2, B-1050 Brussels. Belgium, bart@ai.vub.ac.be
[2] Language and Cognition Department, Max Planck Institute for Psycholinguistics, Wundtlaan 1, 6525 XD Nijmegen, The Netherlands

S.1: Full transition matrices

The full transition matrices $\mathbf{M}_i$, $\mathbf{K}_i$ and $\mathbf{\Lambda}_i$ for the model with two cultural states investigated here are as follows:

$$\mathbf{M}_i = \begin{pmatrix} p(i-1|i,+)\cdot(1-p(-|+)) & p(i-1|i,-)\cdot p(+|-) \\ p(i-1|i,+)\cdot p(-|+) & p(i-1|i,-)\cdot(1-p(+|-)) \end{pmatrix}$$
$$= \begin{pmatrix} \frac{i}{N}\frac{N-i}{N+\varphi\cdot i}\left(1-\frac{\eta}{N}\left(1-\pi\left(\frac{i}{N}\right)\right)\right) & \frac{i}{N}\frac{N-i}{N-\varphi\cdot i}\frac{\eta}{N}\pi\left(\frac{i}{N}\right) \\ \frac{i}{N}\frac{N-i}{N+\varphi\cdot i}\frac{\eta}{N}\left(1-\pi\left(\frac{i}{N}\right)\right) & \frac{i}{N}\frac{N-i}{N-\varphi\cdot i}\left(1-\frac{\eta}{N}\pi\left(\frac{i}{N}\right)\right) \end{pmatrix} \quad \text{(S.1)}$$

$$\mathbf{K}_i = \begin{pmatrix} p(i|i,+)\cdot(1-p(-|+)) & p(i|i,-)\cdot p(+|-) \\ p(i|i,+)\cdot p(-|+) & p(i|i,-)\cdot(1-p(+|-)) \end{pmatrix}$$
$$= \begin{pmatrix} \frac{N-i}{N}\frac{1+(1+\varphi)i}{N+\varphi\cdot i}\left(1-\frac{\eta}{N}\left(1-\pi\left(\frac{i}{N}\right)\right)\right) & \frac{N-i}{N}\frac{1+(1-\varphi)i}{N-\varphi\cdot i}\frac{\eta}{N}\pi\left(\frac{i}{N}\right) \\ \frac{N-i}{N}\frac{1+(1+\varphi)i}{N+\varphi\cdot i}\frac{\eta}{N}\left(1-\pi\left(\frac{i}{N}\right)\right) & \frac{N-i}{N}\frac{1+(1-\varphi)i}{N-\varphi\cdot i}\left(1-\frac{\eta}{N}\pi\left(\frac{i}{N}\right)\right) \end{pmatrix} \quad \text{(S.2)}$$

$$\mathbf{\Lambda}_i = \begin{pmatrix} p(i+1|i,+)\cdot(1-p(-|+)) & p(i+1|i,-)\cdot p(+|-) \\ p(i+1|i,+)\cdot p(-|+) & p(i+1|i,-)\cdot(1-p(+|-)) \end{pmatrix}$$
$$= \begin{pmatrix} \frac{N-i}{N}\frac{(1+\varphi)i}{N+\varphi\cdot i}\left(1-\frac{\eta}{N}\left(1-\pi\left(\frac{i}{N}\right)\right)\right) & \frac{N-i}{N}\frac{(1-\varphi)i}{N-\varphi\cdot i}\frac{\eta}{N}\pi\left(\frac{i}{N}\right) \\ \frac{N-i}{N}\frac{(1+\varphi)i}{N+\varphi\cdot i}\frac{\eta}{N}\left(1-\pi\left(\frac{i}{N}\right)\right) & \frac{N-i}{N}\frac{(1-\varphi)i}{N-\varphi\cdot i}\left(1-\frac{\eta}{N}\pi\left(\frac{i}{N}\right)\right) \end{pmatrix} \quad \text{(S.3)}$$

S.2: Fixation graphs for starting in the dispreferred state

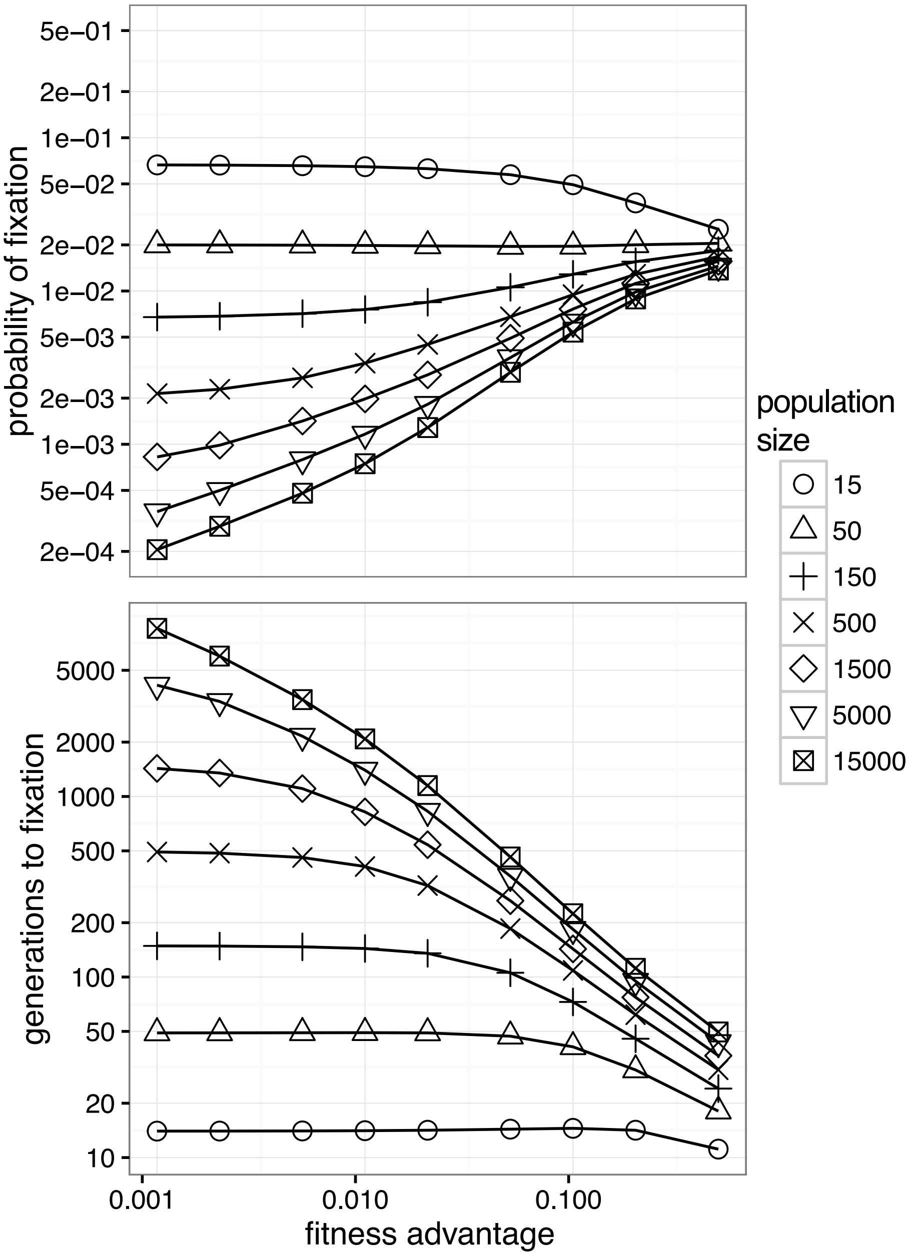

Figure S.1: Fixation probabilities and times for varying population sizes and fitness differences when the population starts in the dispreferred cultural state. Parameter settings are the same as in figure 3.

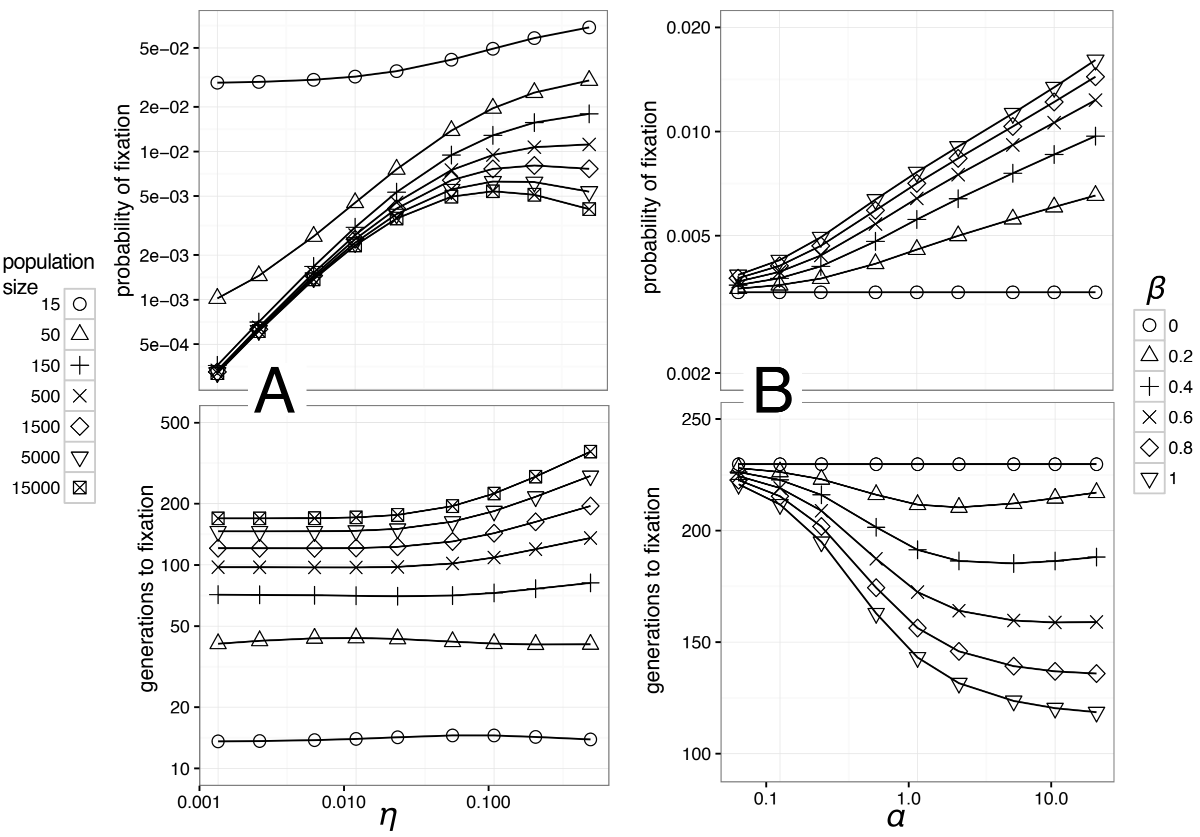

Figure S.2: Absolute fixation probabilities and times for when evolution starts in the dispreferred cultural state. Parameter values are the same as in figure 4.

S.3: Analysis of the model of Chater et al. 2009

The model described by Chater *et al*. 2009 implements evolution in a very different way than the Moran process: a new generation of individuals is determined in one step, not individual by individual. Individuals are ordered on the basis of a (stochastic) quality criterion, and a pre-determined fraction of the best individuals is allowed to reproduce. The original population size is then restored by a (uniform) random selection of these individuals. New individuals are formed by crossing two individuals, but because the model described in the present paper only looks at the evolution of a single trait, crossover can be ignored. The result of these choices (which appear to be mostly based in the tradition of computational genetic algorithms) is that the probability of a trait to end up in the next generation depends in a complicated way not just on the composition of an individual, but also on the composition of other individuals in the population.

Nevertheless, it is possible to get an impression of the impact of mutations from a generalist to a specialist if some assumptions are made about population composition. Because the focus of this paper is on the probability of a population of generalists being taken over by specialists (and the time that takes) an initial population in which all agents have a generalist allele at a given position (the target allele) will be assumed. The other genes are selected at random with equal probabilities from the three possibilities in the CRC model (specialist in the two possible cultural states or generalist). The parameters are identical to the default parameters in Baronchelli *et al*. (2013). The number of genes per individual $L = 20$, the proportion of individuals that goes to the next generation $f = 0.5$, and the parameter that determines the effect of being a specialist, $p = 0.95$. The parameter p determines the probability that an individual guesses the parameter of the language correctly in each learning step. The expected number of learning steps then follows a geometric distribution with a mean number of steps of $1/p \approx 1.05$ for specialists in the preferred state. For a specialist in the dispreferred state this is chosen to be $1/(1-p) = 20$. For generalists the mean number of steps is equal to $1/0.5 = 2$.

In order to estimate the probabilities of having offspring in the next generation for specialists in the preferred and dispreferred states, one million random individuals were generated for three different cases: one in which the target gene was the generalist variant, one in which it was the specialist variant in the dispreferred cultural state and one in which

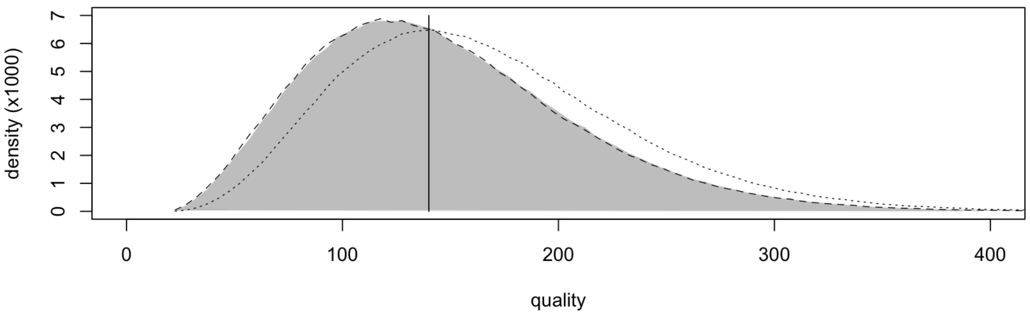

Figure S.3: Estimated density functions of the learning time of individuals with a generalist target allele (grey area) with a preferred specialist target allele (dashed line) and with a dispreferred specialist target allele (dotted line). The vertical line indicates the median value of the learning times in the generalist case. In a population of generalists, only individuals with learning times below this value would be selected for the next generation. Note that the disadvantage of specialists in the dispreferred cultural state is much larger than the advantage of specialists in the preferred state.

it was the specialist variant in the preferred cultural state. For each individual the number of steps it would take to learn the language is randomly generated (because the learning process in the CRC model is random, learning times are random values). Histograms for the three cases as well as the median value of the total learning time for the generalist value of the target gene are shown in figure S.3.
It can be observed that the distribution of learning times is almost the same for individuals with a specialist target allele in the preferred cultiural state and for individuals with a generalist target allele. However, individuals with a specialist target allele for the disprefewrred cultural state have a much longer learning time. The median value of individuals with a generalist target allele is 140, that of individuals with a specialist target allele in the preferred cultural state is 139 and that of individuals with a specialist target allele in the dispreferred cultural state is 158. This is as expected from the mean learning times mentioned above. As for the probabilities of ending up in the next generation (starting with a population where every individual has the generalist target allele) is 50.5% for the individuals with the specialist target allele in the preferred cultural state. It is 38.3% for individuals with the speciaist target allele in the dispreferred cultural state. Thus the fitness disadvantage for specialists is about 20 times higher than the fitness advantage. As there is some randomness in these estimates, which causes an especially large uncertainty for the small advantage of the specialists in the preferred cultural state, which may range from 0.3%-0.7%, a somewhat more conservative ratio of 1:16 was therefore used for the calculations.

The effect of the much larger disadvantage of being in the dispreferred cultural state than the advantage of being in the preferred cultural state is shown in figure S.4. It is estimated that a fitness disadvantage of about 0.1 is equivalent to what happens in Chater et al.'s (2009) model with the parameters described above. The fitness advantage is 1/16th of this value (0.00625). For a population of 150 individuals (comparable to their population sizes) this results in an approximately 40 times smaller probability of fixation than in the case where there is no cultural change. Given that in the case were the fitness advantage equals the fitness disadvantage, the fixation probability is only twice as small for these parameter values (they can be found in figure 3) it is clear that in Chater et al.'s (2009) model specialists have an inherent disadvantage compared to generalists. For larger populations the difference is even more dramatic.

The fixation times are not much longer, but it should be kept in mind that these are conditional fixation times, i.e. the time it takes for the population to reach fixation, under the condition that the specialists take over from the generalists.

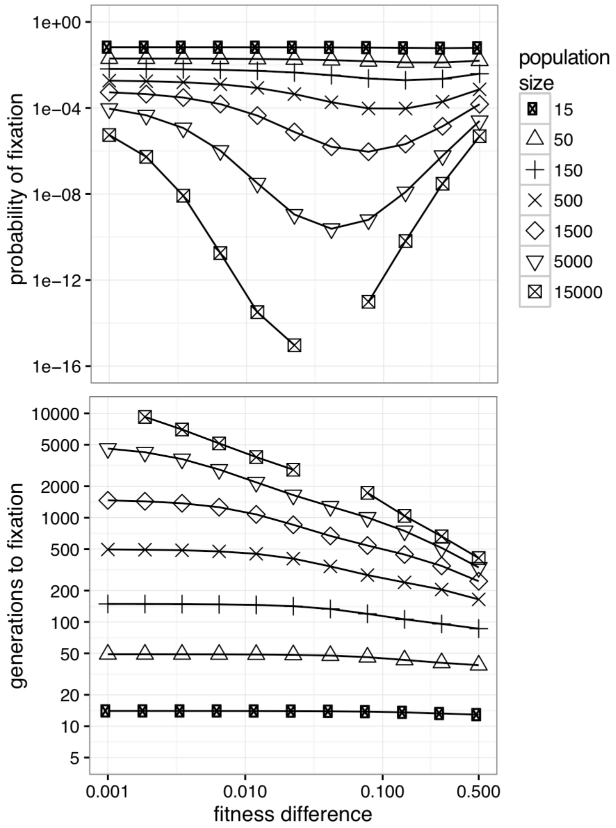

Figure S.4: Fixation probabilities and fixation times when specialists have a 16 times higher fitness disadvantage than a fitness advantage. The matrix solver did not converge for fitness disadvantage 0.0026 and population size 15 000. It is estimated that a fitness disadvantage of approximately 0.1 corresponds to Chater et al.'s (2009) model. Other parameters are: $\alpha = \beta = 1$, $\eta = 0.1$

S.4 Fixation graphs showing relative probability and time

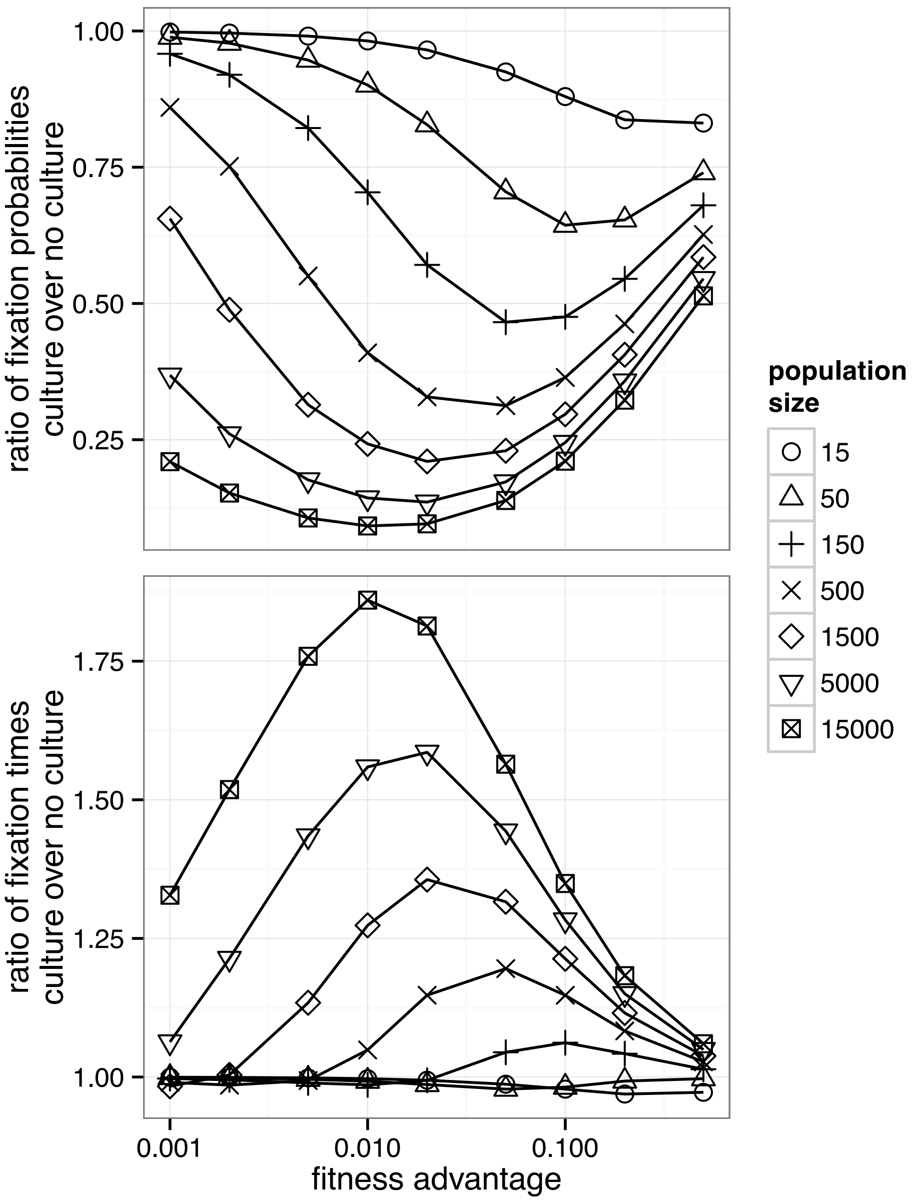

Figure S.5: Fixation probabilities and fixation times of systems with culture, relative to systems without culture, starting in the preferred state. Parameter settings are the same as in figure 3.

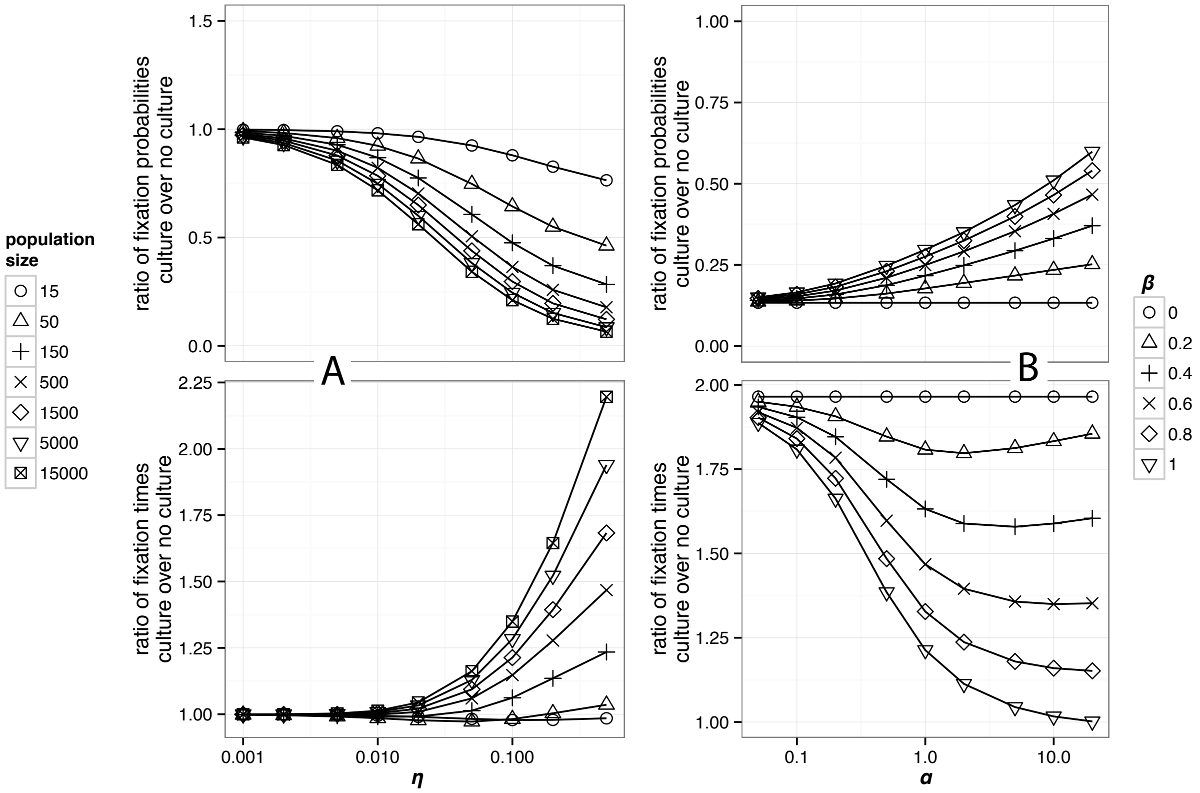

Figure S.6 Fixations for different variants of cultural parameters relative to the situation without cultural change, starting in the preferred state. Parameter values are the same as in figure 4.

References

Baronchelli, A., Chater, N., Christiansen, M. H., & Pastor-Satorras, R. (2013). Evolution in a changing environment. *PloS One*, *8*(1), e52742.

Chater, N., Reali, F., & Christiansen, M. H. (2009). Restrictions on biological adaptation in language evolution. *Proceedings of the National Academy of Sciences*, *106*(4), 1015–1020.
